# 3D visualization of the human anterior cruciate ligament combining micro-CT and histological analysis

**DOI:** 10.1007/s00276-023-03295-5

**Published:** 2024-01-24

**Authors:** Annapaola Parrilli, Alberto Grassi, Federica Orellana, Roberta Lolli, Gregorio Marchiori, Matteo Berni, Milena Fini, Nicola Francesco Lopomo, Stefano Zaffagnini

**Affiliations:** 1 Center for X-Ray Analytics, Empa - Swiss Federal Laboratories for Materials Science and Technology, Überlandstrasse 129, 8600 Dübendorf, Switzerland; 2 IRCCS - Istituto Ortopedico Rizzoli, Bologna, Italy; 3 University of Fribourg, Fribourg, Switzerland; 4 University of Brescia, Brescia, Italy

**Keywords:** ACL, Micro-CT, Human knee, 3D anatomy, Soft tissue X-ray imaging, Anterior cruciate ligament

## Abstract

**Purpose:**

The study aimed to obtain a comprehensive 3D visualization of knee specimens, including the cruciate ligaments and corresponding femoral and tibial bone insertions using a non-destructive micro-CT method.

**Methods:**

Knee specimens were fixed in anatomical positions and chemically dehydrated before being scanned using micro-CT with a voxel size of 17.5 μm. RGBA (red, green, blue, alpha) transfer functions were applied to virtually colorize each structure. Following micro-CT scanning, the samples were rehydrated, decalcified, and trimmed based on micro-CT 3D reconstructions as references. Histological evaluations were performed on the trimmed samples. Histological and micro-CT images were registered to morphologically and densitometrically assess the 4-layer insertion of the ACL into the bone.

**Results:**

The output of the micro-CT images of the knee in extension and flexion allowed a clear differentiation of the morphologies of both soft and hard tissues, such as the ACL, femoral and tibial bones, and cartilage, and the subsequent creation of 3D composite models useful for accurately tracing the entire morphology of the ligament, including its fiber and bundle components, the trajectory between the femur and tibia, and the size, extension, and morphology of its insertions into the bones.

**Conclusion:**

The implementation of the non-destructive micro-CT method allowed complete visualization of all the different components of the knee specimens. This allowed correlative imaging by micro-CT and histology, accurate planning of histological sections, and virtual anatomical and microstructural analysis. The micro-CT approach provided an unprecedented 3D level of detail, offering a viable means to study ACL anatomy.

**Supplementary Information:**

The online version contains supplementary material available at 10.1007/s00276-023-03295-5.

## Introduction

Orthopedic surgeons have always considered a comprehensive knowledge of both the gross and detailed morphology of the anterior cruciate ligament (ACL) as the basis for a biomechanically valid reconstruction [10]. Despite this clinical need, the anatomy of the ACL remains a controversial topic that defines a very active field of research [9, 24, 28]. To date, there is disagreement in the literature regarding the identification of both the morphological structure of the mid-substance (double bundle or ribbon) [18, 23] and the shape of the ACL insertion footprint in the tibial or femoral bone segments [11].

Most anatomical three-dimensional (3D) studies focused on the ACL are based on macroscopic evaluations [2] or on the use of magnetic resonance imaging (MRI) [26, 29]. On the other hand, the use of computed tomography (CT) is limited to the morphological evaluation of the bony components of the joint to define critical bony landmarks such as the resident’s ridge [15]. However, these qualitative methods for morphological assessment have some inherent limitations, including the resolution of clinical imaging techniques (e.g., CT and MRI) limited to 200–300 μm in voxel size, operator dependence on the accuracy of specimen dissection in macroscopic analysis, and low radiographic contrast of soft tissues in radiographic analysis. Several authors have proposed the use of different 3D visualization techniques in conjunction with histological analysis [20]. Indeed, histology is the most valuable technique for visualizing the 4-layer structure of the direct insertion of the ACL into bone, specifically consisting of ligamentous tissue, non-calcified fibrocartilage, calcified fibrocartilage, and bone. However, due to its inherent two-dimensional (2D) nature, histological analysis is limited by the number of sections obtainable for each sample and by the process of spatially aligning these sections required for comprehensive 3D localization.

In contrast, micro-computed tomography (micro-CT) is a well-established technology in non-clinical research and today represents one of the most used for 3D visualization and quantitative analysis [25]. In particular, micro-CT is currently and widely used in orthopedic preclinical studies [6, 8, 22]. As for clinical CT, the 3D visualization of soft tissues (alone or in combination with hard tissues) is still a challenge. The microstructural visualization of these tissues with low X-ray absorption requires the use of contrast agents with high z-element staining [14, 19]. Nonetheless, the utilization of contrast agents may give rise to certain challenges, encompassing possible tissue harm and the selectivity of such agents. These factors can compromise the precise interpretation of outcomes and their potential for comparison and reproducibility [7]. In different research fields, such as zoology, the micro-CT evaluation of gross morphology and tissue arrangement has been achieved by using a critical drying point protocol, even without the introduction of contrast agents [4, 21].

The aim of this study was to implement a micro-CT method to obtain 3D visualization of the human ACL without the use of contrast agent staining. Our main hypothesis was based on the possibility of highlighting ligament morphology and tissue arrangement using only a chemical drying protocol. The method did not invalidate the non-destructive nature of micro-CT analysis, allowing subsequent histology to be performed on the same samples. Human knee specimens, including cruciate ligaments and central portions of the bony structures, were analyzed in flexion and extension positions, while histologic evaluation was performed according to the axial or sagittal plane of sectioning.

## Materials and methods

### Sample preparation

**Fig. 1:**
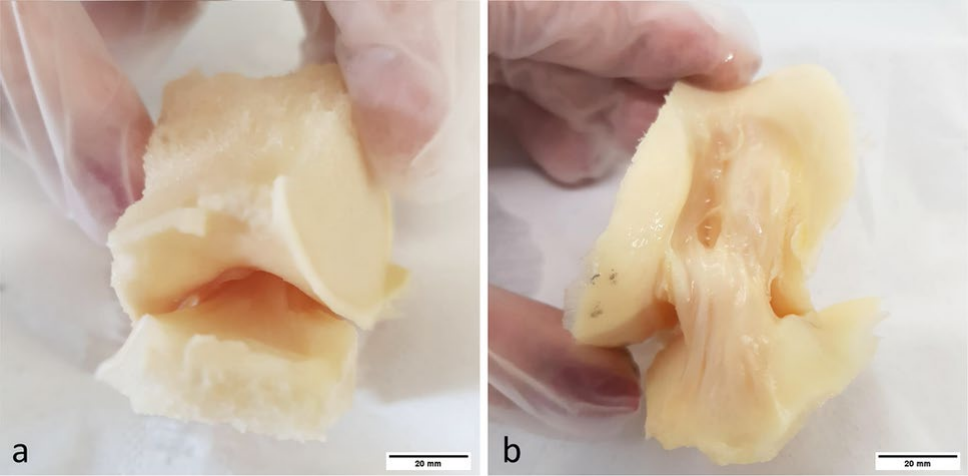
Human knee joint specimens were used for direct 3D micro-CT visualization of the anatomy of the ACL. The specimens included the anterior and posterior cruciate ligaments, the central portion of the distal femur (intercondylar notch, medial border of the lateral femoral condyle, and lateral border of the medial femoral condyle), and the central portion of the tibial plateau (nearly 1 cm in height). The specimens were held at knee extension (**a**) and at knee flexion (**b**). *Scale bars* 20 mm

Two human ACLs were obtained from the same healthy donor (female, 60 years old). Our study was performed in accordance with the European and Italian laws for cadaveric studies. The research protocol was approved by the ethics committee of the Rizzoli Orthopedic Institute (protocol n°0008425; approved: 31/08/2017). The specimens included the intercondylar notch with the ACL, the posterior cruciate ligament (PCL), the medial border of the lateral femoral condyle, the lateral border of the medial femoral condyle, and 1 cm of the central part of the tibial plateau. No dissection was performed. Using simple needles threaded through the femoral and tibial bone segments, one specimen was held at knee flexion and the other at knee extension (Fig. 1). The specimens were fixed in 10% neutral buffered formalin for 48 h to stabilize the tissue components. After washing in distilled water for 24 h, the samples were dehydrated in 70% ethanol overnight, 80% ethanol for 3 h, 90% ethanol for 3 h, 100% ethanol overnight. To enable three-dimensional visualization of the specific region within the human knee joint, with a particular emphasis on the ACL, a chemical drying procedure was employed placing the specimen in hexamethyldisilazane for 2 h. The samples were then air dried in a fume hood for at least 12 h. The procedure was performed on both specimens in knee flexion or extension.

### MicroCT analysis

The dry specimens were scanned using the Skyscan 1176 micro-CT system (Bruker micro-CT, Kontich, Belgium) with a source voltage of 45 kV, source current of 550 μA, an aluminum filter of 0.2 mm, and an exposure time of 185 ms. Each sample was rotated 180° with a rotation interval of 0.2° and a frame average of 5 to reduce the signal-to-noise ratio and improve image quality. The voxel size was 17.5 μm. Images were reconstructed using NRecon software (version 1.6.10.4) with corrections for specific misalignment of each acquisition, low ring artifact reduction, and beam hardening. The micro-CT image datasets contained 3800 slices (4000 × 4000 pixels) for each sample. 3D models of both knees in flexion and extension positions and of all their components (bone, ACL and PCL) were generated using CTAn and CTVox software. In order to distinguish the different components of the knee joint (bone, ACL, PCL and cartilage) and to enhance details (e.g. fibrous structure of the ligaments), we applied RGBA (red, green, blue, alpha) transfer function adjustment to the virtual coloring of the 3D models.

### Histological assessment

After scanning, the samples were rehydrated with the same graded series of ethanol in reverse order and washed in distilled water for 6 h. The samples were then decalcified with a solution of 5% formic acid (50 ml) and 4% nitric acid (40 ml) in 1 l of distilled water, adjusting the proportions to obtain the desired volumes. The samples were left in this decalcifying solution for two weeks, but since the process is dependent on sample size, we monitored the softness of the bone tissue in the meantime. After decalcification, the samples were placed in distilled water for 24 h. Before being processed for paraffin embedding, they were accurately trimmed with a scalpel in different portions using micro-CT 3D models as a reference to fit them into tissue cassettes. In addition, the micro-CT 3D models were used as a guide to determine the orientation of the tissue. The paraffin embedding protocol consisted of dehydrating the specimens in 70% ethanol for 24 h, 95% ethanol for 2 h, 100% ethanol for 3 h, xylene for 2 h, and embedded in paraffin.

Then both hematoxylin and eosin (H&E) and Safranin O staining protocols were used. In order to allow a full insight of the samples, we cut the samples along two different spatial planes and obtained 5 µm thick sections: for the sample kept at knee extension, the histological cut was performed according to the axial anatomical plane, while for the sample held in knee flexion, the histological cut was performed according to the sagittal anatomical plane.

### MicroCT and histology imaging comparison

Nearly 10 histological Sects. (5 H&E and 5 safranin O) of each sample were spatially localized using as reference the 3D microtomographic datasets displayed in orthogonal projection on the three spatial planes with Dataviewer software (Bruker micro-CT, Kontich, Belgium). The micro-CT 3D models served as valuable references for paraffin embedding and sectioning, allowing precise 3D spatial localization of the histological sections. In addition, two histological sections were registered with the corresponding micro-CT sections in order to overlap the structural features obtained with the two different techniques and to compare them with their real 3D anatomical position in the 3D models. To achieve the overlay, we incorporated the alpha channel to preserve image transparency and removed the background from the digital histological sections. This meticulous process allowed for precise alignment of the histological images with the corresponding micro-CT sections.

## Results

### Micro-CT acquisition of human ACL using chemical drying

**Fig. 2:**
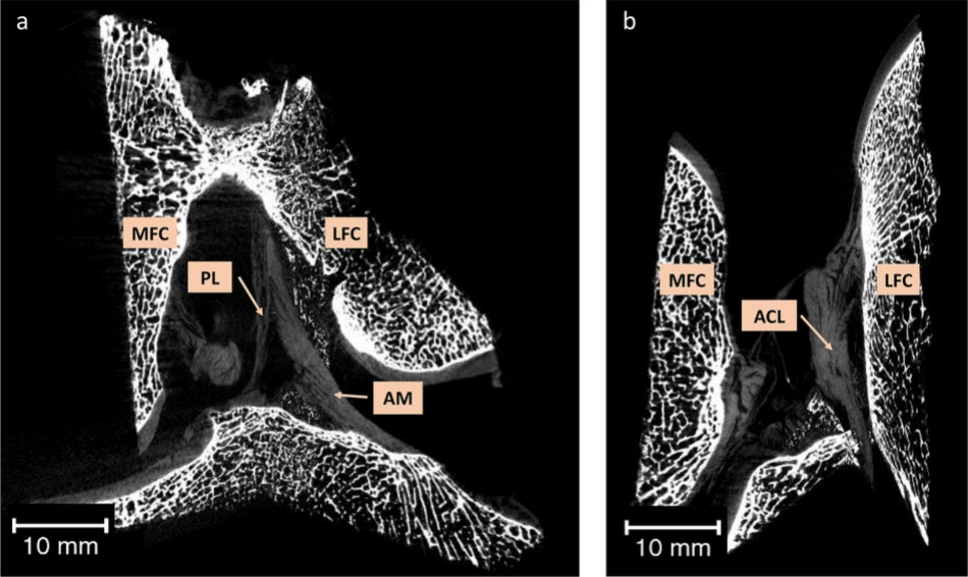
Coronal micro-CT 2D sections of the human knee joint specimen with anatomical references. **a** 2D section of the specimen held in knee extension. **b** 2D section of the specimen held in knee flexion. *MFC* medial femoral condyle, *LFC* lateral femoral condyle, *ACL* anterior cruciate ligament, *AM* anteromedial bundle of the ACL, *PL* posterolateral bundle of the ACL. *Scale bars* 10 mm

The micro-CT imaging analysis allowed the visualization of the ACL’s structure and morphology within the knee joint. The analysis were performed for specimens held in knee extension (Fig. 2a) and flexion (Fig. 2b).

**Fig. 3:**
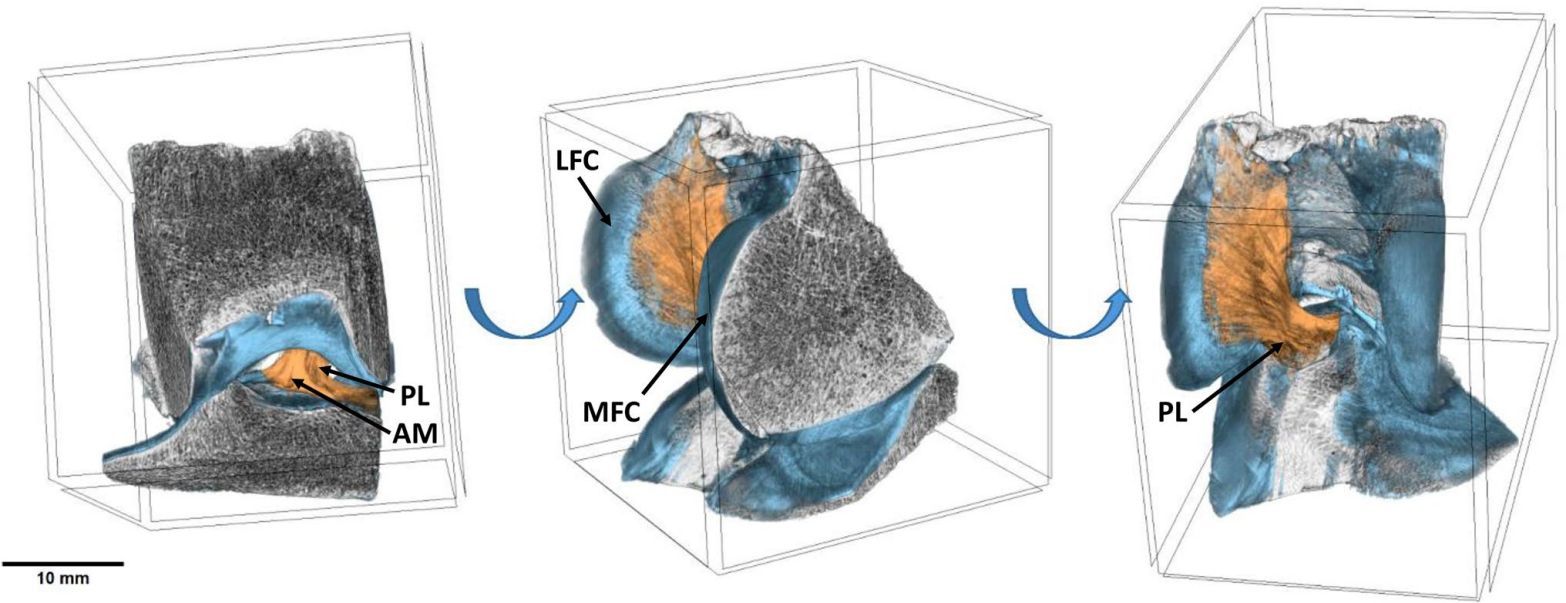
3D model of the human knee joint specimen held in extension position. The model is shown from different angles (anterior, lateral and posterior perspectives from left to right in the image). Bone is colored *gray*, cartilage is colored *blue* and ACL is colored *orange*. *MFC* medial femoral condyle, *LFC* lateral femoral condyle, *AM* anteromedial bundle of the ACL, *PL* posterolateral bundle of the ACL. *Scale bar* 10 mm (color figure online)

The processing protocol preserved the distinct X-ray absorption properties of the various components within the joint. As a result, these different components could be easily identified and digitally separated for subsequent 3D modeling. In fact, separate structures such as ACL, bone (femur and tibia), and cartilage have been identified in micro-CT section datasets both by their different morphology and by contrast imaging output. Each of these distinct tissues were individually colored, leading to the creation of 3D models after the micro-CT analysis process (Fig. 3).

**Fig. 4:**
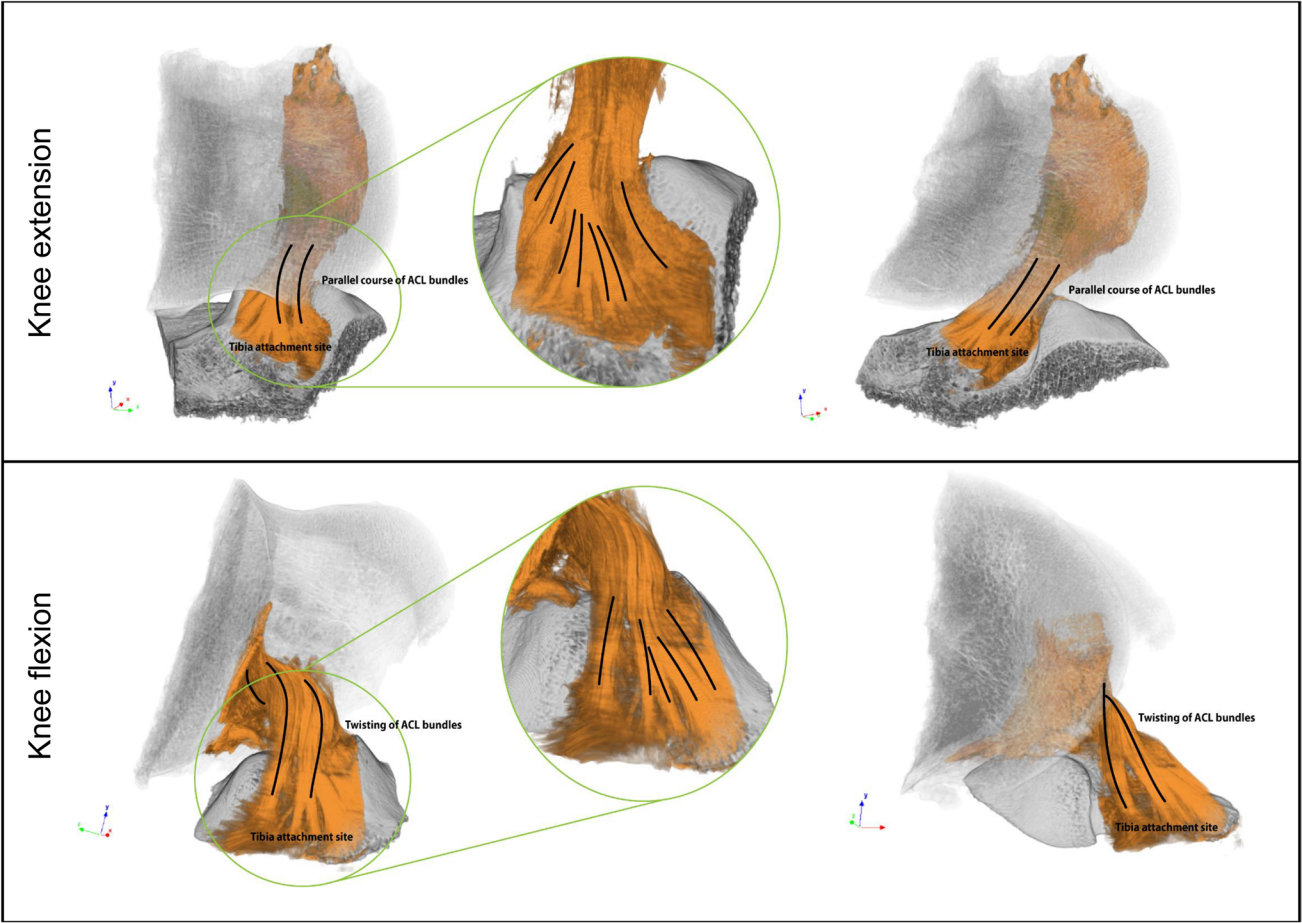
Micro-CT 3D models of the specimens held in knee extension and flexion. Bone is colored *gray* and ACL is colored *orange*. The *inset* shows a magnification of the tibial attachment site (color figure online)

In the 3D micro-CT models, the ACL is organized into multiple parallel-oriented bundles in the extended knee, whereas in the flexed knee, torsional movement of the bundles disrupts this parallel orientation. In both positions, the bundles are firmly anchored to the tibia and form a roots-like structure (Fig. 4).

A comprehensive 3D video (see Online Resource Video 1) was generated demonstrating the reconstruction of ACL anatomy using microCT analysis. The video employs techniques such as the gradual rendering of certain knee joint components transparent or opaque, virtual slicing of the model, and navigation within the model to provide a detailed description of the real ACL fiber structure as it evolves along its 3D path from tibial to femoral insertion.

### Direct comparison of micro-CT analysis with histology

**Fig. 5:**
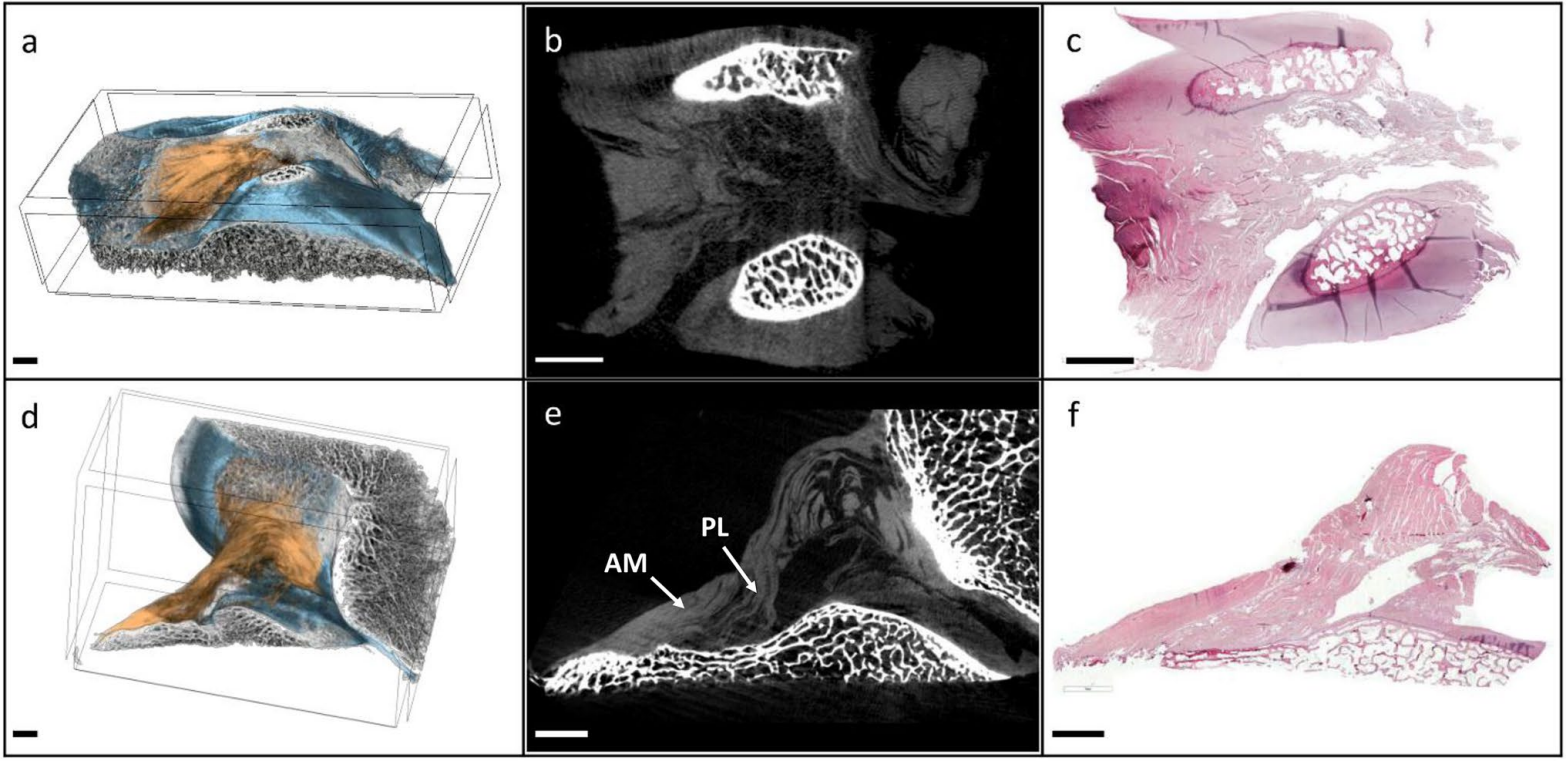
Direct comparison of micro-CT 3D models and 2D sections with histology. **a** 3D model of the ACL insertion at the tibial level of the specimen held in knee extension, virtually sectioned along the anatomical axial plane; bone is stained *gray*, cartilage is stained *blue*, and ACL is stained *orange*. **b** Corresponding micro-CT 2D section of model (**a**). **c** Corresponding histological section stained with H&E of model (**a**) and micro-CT section (**b**). **d** 3D model of the ACL insertions at the tibial and femoral levels of the specimen held in knee flexion, sliced along the anatomical sagittal plane, colors are the same as the model in (**a**); **e** corresponding micro-CT 2D section of model (**d**). **f** Corresponding histological section stained with H&E of model (**d**) and micro-CT section (**e**). *AM* anteromedial bundle of the ACL, *PL* posterolateral bundle of the ACL. *Scale bars* 3 mm (color figure online)

The use of different sectioning planes in histology and the precise localization of each histological section with respect to its spatial location and position with respect to the 3D model allowed direct comparisons with the corresponding microtomographic images (Fig. 5).

**Fig. 6:**
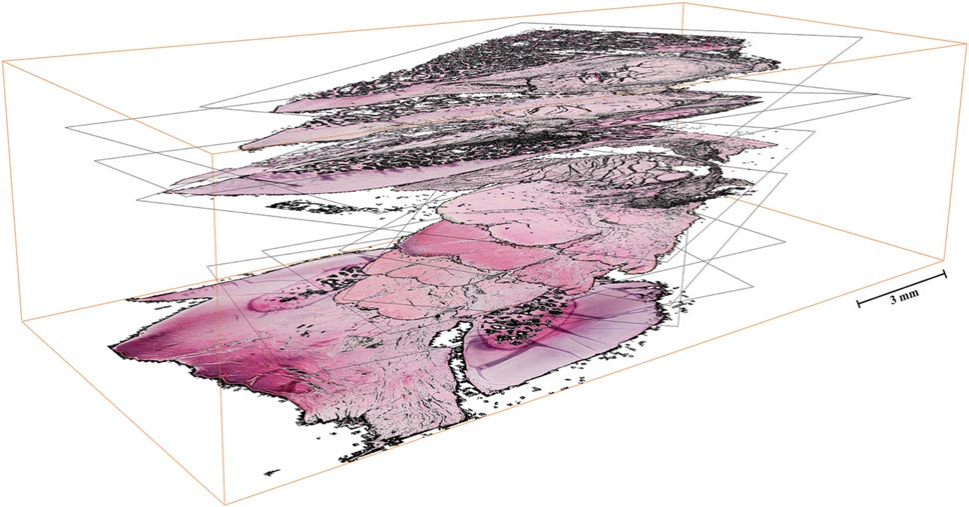
Virtual 3D representation of the ACL path from the tibia to the femoral insertion. Arbitrary numbers of H&E stained histological images were stacked according to the approximate alignment known from micro-CT models. Histologic images refer to the ACL of the specimen held at knee extension, sectioned along the anatomic axial plane from the tibial insertion (*bottom*) to the femoral insertion (*top*). *Scale bar* 3 mm

Following the outlined evaluation design, a pseudo-three-dimensional image was created by stacking different histological sections of the sample maintained at full knee extension. These sections were cut along the axial anatomical plane (Fig. 6). The construction of this pseudo-three-dimensional image involved sequentially arranging the individual histologic images to simulate a three-dimensional representation. Each of the individual images forming the 3D histological approximation (as shown in Fig. 6) can be readily identified in the three-dimensional model generated by micro-CT analysis, similar to the corresponding representation in Fig. 5a–c.

**Fig. 7:**
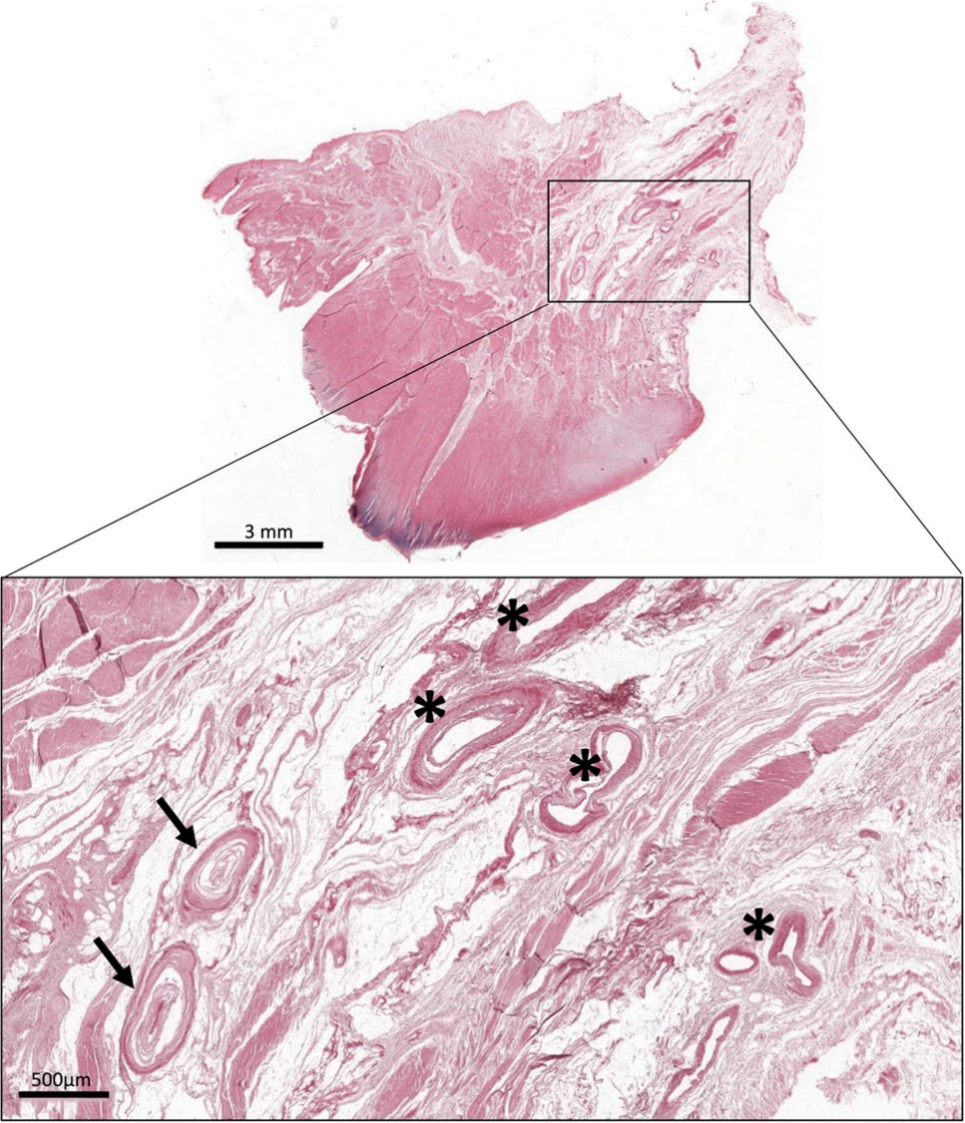
Morphological appearance of vascular and neural structures. Free nerve endings (*arrows*) and periligamentous vessels (*asterisks*) were identified within the ACL in the histological sections stained with H&E

Despite the limitations and invasiveness of the protocol on the specimens, it proved to be suitable for defining the anatomy of some important structures within the joint. In fact, blood vessels and nerves could be confirmed in the histological sections (Fig. 7).

### Histology and micro-CT 2D image registration and relative densitometric observations of the 4-layer structure of the ACL insertion

**Fig. 8:**
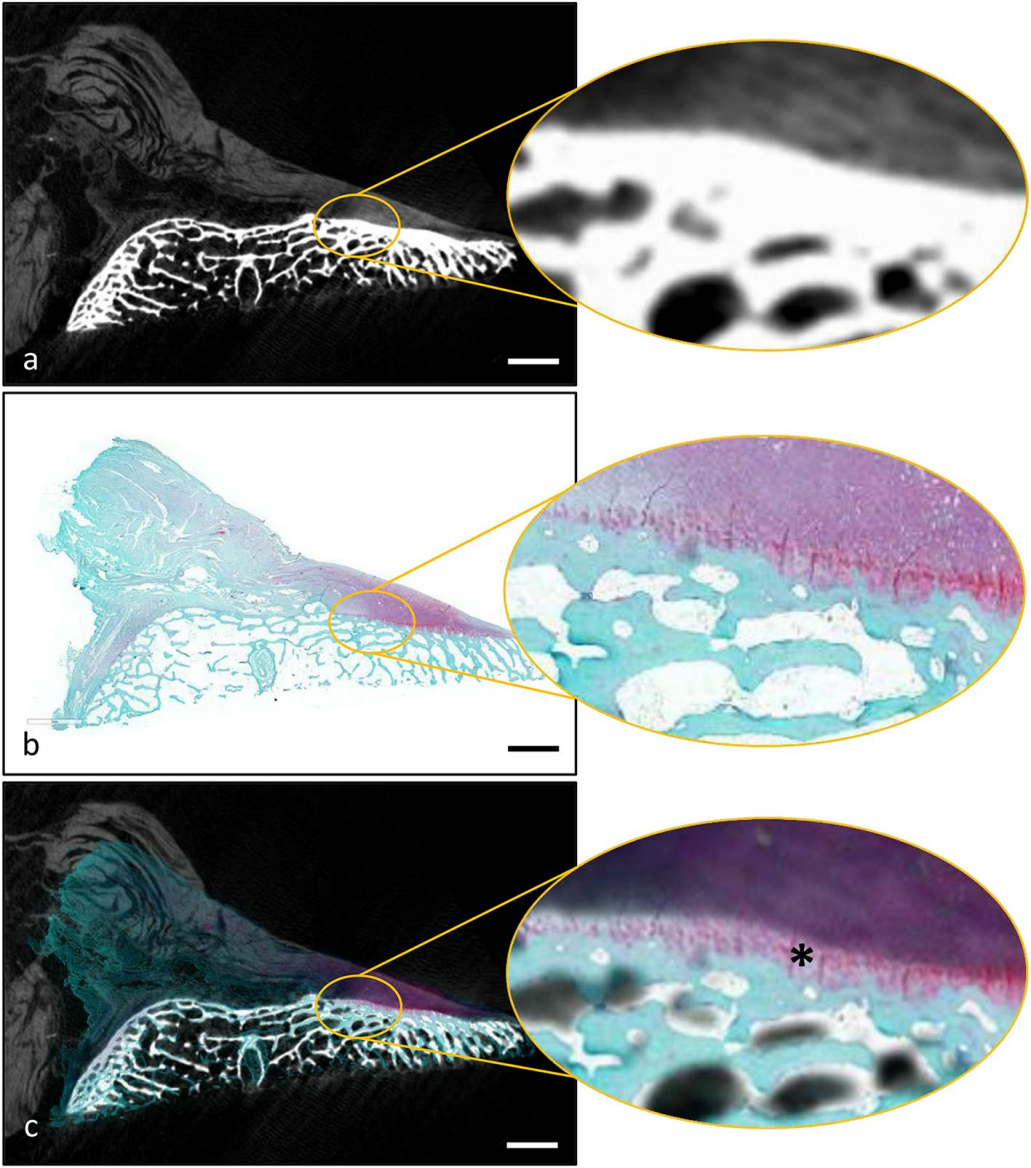
2D image registration of histological and micro-CT sections of the ACL. **a** Micro-CT section of the ACL insertion at the tibial level of the specimen held at knee flexion, virtually sectioned along the anatomical sagittal plane. The *inset* shows a magnification of the insertion area. **b** Histologic section and its magnification in the relative inset shown at the same spatial level of the micro-CT image above. **c** The overlay of the two images. The *asterisk* in the inset shows the calcified fibrocartilage layer of the ACL insertion in the tibia. *Scale bars* 3 mm

In the micro-CT sections, the identification of different components relies on their distinct X-ray absorption levels. Consequently, the grey levels in digital micro-CT images primarily reflect the chemical composition of the sample components. As a result, areas with higher mineralization exhibit pixels at lighter grey levels compared to those containing soft tissues or the background. Conversely, in histological images, safranin O staining is employed. The bone component appears green due to the Fast Green dye, while at the ACL insertion level, the fibrocartilage (both calcified and non-calcified) appears in a bright pink/violet color due to the safranin dye. Thus, each technique (micro-CT and histology), taken individually, identifies only one characteristic of the fibrocartilaginous component in the transition zone. The ability to obtain sections of the same sample using different techniques facilitated 2D image registration (Fig. 8). By overlaying images from sections cut at the same spatial level, a precise identification of the calcified fibrocartilage area was achieved. This was made possible by the accurate matching of pink-colored pixels from histological sections representing the fibrocartilaginous zone with the bright light grey levels of pixels from micro-CT sections representing the mineralized zone (Fig. 8c).

## Discussion

In this study, we developed and applied a micro-CT analysis protocol to achieve comprehensive 3D visualization of the entire knee joint components, including both soft and hard tissues, without the need for contrast agents. Using chemical drying, we successfully examined and visualized the superficial and internal morphology of the human knee in a non-destructive and three-dimensional manner. ACL morphology was thoroughly evaluated in two knee positions: extension and flexion, allowing identification and visualization of bundles and 3D insertions within the bone. This approach provided valuable insight into how the conformation of the ACL bundles corresponds to their physiological activities. The 3D data sets allowed detailed images and videos of the ACL anatomy through 3D reconstruction, facilitating virtual macroscopic analysis of the spatial configurations of different joint components, in particular the shape of ACL insertions at both the femoral and tibial levels. From a clinical point of view, it is indeed fundamental to describe the real evolution of the ACL fibrous structure along its 3D path in the knee joint. The inherent advantages of three-dimensional visualization have significant clinical and educational value, especially when operators can interact directly with the model or when these interactions are presented in explanatory videos (see Online Resource Video 1).

The approach of using the entire knee specimen without the need for prior tissue identification allowed for the avoidance of soft tissue dissection. This preservation of anatomical integrity was particularly important for the fibrous component of the ligament and its original path between the femur and tibia. In previous studies based on macroscopic anatomical classification rely on dissection of soft parts by anatomo-pathologist preserving major components but potentially damaging the complex fibrous network. In any case, when comparing the results of our study with those of previous investigations of ACL anatomy, several notable similarities and differences emerged. Our study confirms the established anatomical structure of the double-bundle ACL, with clear visualization of its fiber and bundle components, as well as its insertions into the femur and tibia. For example, we observed ACL insertion at the level of the femur with a characteristic appearance of fan-like extension both anteriorly and posteriorly along the lateral surface of the intercondylar notch, also observed by Mochizuchi et al. [16]. Furthermore, similar to other studies that have emphasized the functional differences between the AM and PL bundles [5] in our 3D visualizations the two bundles are easily identifiable, especially in flexion position and at the height of the insertion to the tibia. An important aspect that emerged from the highlighting of the fibrous component by chemical dehydration was the observation of the more branched and root-like appearance of the bundles at the tibial insertion, especially in the AM. To our knowledge, this type of conformation has never been detailed in previous studies although it is still described generically as fanning out to form the foot-region [23, 28].

Similarly with other studies [12, 16] we observed an attachment area of the AM bundle larger than that of the PL bundle. During knee extension, the PL bundle is tense, while the AM bundle is moderately relaxed. Knee flexion prompts a shift in the ACL’s femoral attachment, causing the AM bundle to tighten and the PL bundle to relax [23]. Given this aspect of the double bundle conformation, we observed the classic twisted configuration with external twisting of the fibers when the knee is flexed due to the relative alignment of the bony insertions. This agreement with previous research underscores the consistency and reliability of anatomical descriptions of the ACL using non-destructive micro-CT which also allows simultaneous visualization of soft and hard tissue structure with micrometer resolution.

Understanding the double bundle anatomy of the ACL is essential for customizing precise anatomical reconstructions [12]. Functionally, the AM bundle primarily combats anterior tibial translation, whereas the PL bundle contributes to knee stability during extension and safeguards against rotational stresses, especially near full extension [23]. Future study using this technique on a larger sample size could give broader indications with respect to new, more realistic and therefore more functional surgical reconstruction strategies. In addition, since the major causes of ACL reconstruction failure are due to errors during the initial ACL reconstruction surgery [13] or to underlying biological influences [27], as well as to subsequent trauma [1, 30], this study opens the possibility for a more in-depth examination of the complications or poor outcomes of certain techniques or category of patients.

The prospect of overlaying histological images onto micro-CT sections represents a crucial aspect of our investigation. Micro-CT can differentiate two areas based on their degree of mineralization but cannot distinguish between bone and mineralized fibrocartilage using the settings and resolution of this study. On the other hand, histology clearly differentiates between the fibrocartilaginous component and the bone, but the exact identification of the tidemark between the non-calcified and calcified cartilage, although each zone has its specific morphological and mechanical properties, proves challenging and presents a blurred appearance [3]. The 2D registration technique allows for the evaluation of distinctive features that, when combined, enhance our understanding of the morphological and structural aspects of the analyzed sample. Specifically, we can utilize this approach to study the intricate structural characteristics of the ACL at its insertion level from a densitometric perspective, by superimposing histological and micro-CT images. Recently, Muro et al. [17] described through macro anatomical analysis and with serial histologic slicing, the possible insertion of the ACL into both the tibial cartilage and the lateral meniscus. Although the specimens analyzed in our study did not include the menisci and that the X-ray absorption of the involved dehydrated soft tissue (ACL, PCL and cartilage) is similar, we believe that future studies combining serial slicing histology with our microCT analysis procedure may provide more in-depth clarification of the ACL attachment system at the tibial level.

The acquisition of nearly 4000 micro-CT sections per sample at a nominal resolution of 17.5 μm provided a wealth of 3D information that can lead to further extensive analysis and a robust statistical approach. The micro-CT-derived 3D models also served as a reference for sample size reduction and orientation planning for histology. The virtual sectioning capability of the models facilitated precise and accurate histological sectioning and localization of stained histological sections within the actual 3D knee anatomy. This image registration approach and accurate spatial positioning overcame the loss of 3D information in histologic evaluation due to sectioning distance.

Through micro-CT sections, we successfully visualized clear boundaries between the bone tissue and the fibrous ligament tissue. This distinction was achievable due to the differential absorption of X-rays by hard and soft tissues, resulting in varying contrast and gray levels of pixels in the images. Consequently, mineralized and non-mineralized areas could be easily identified and quantified based on specific gray level thresholds. Histologically, the transition zone of ligament attachment consists of four layers: ligament, fibrocartilage, mineralized fibrocartilage and bone. Images of histological sections showing microstructural biological features and micro-CT sections showing microstructural densitometric properties highlighted these gradual changes in stiffness at the ACL-bone junction level. The appropriate degree of transparency of the digital histologic sections was used to overlay the image with the corresponding micro-CT sections.

A key advantage of combining micro-CT imaging with histology is the ability to study a stack of multiple images in three planes. This comprehensive approach provides a more detailed understanding of the complex anatomical structures. In addition, the information obtained from micro-CT analysis can guide and inform the histological examinations, enhancing the accuracy and efficiency of the overall investigation.

The use of 3D visualization further facilitates a comprehensive view of the internal and external aspects of the knee joint structures, with particular emphasis on the ACL. However, we must acknowledge a limitation associated with the use of an invasive drying protocol that may result in potential tissue shrinkage and deformation. Proper quantification and consideration of these effects is essential for accurate volumetric analysis and interpretation of the data obtained.

## Conclusions

Our study demonstrates that micro-CT technology is a viable and valuable tool for investigating the complex anatomy of the ACL. The chemical drying protocol we proposed successfully allowed 3D visualization of the ACL fiber bundle structure, accurate assessment of its shape and evaluation of its insertions into the femur and tibia in both knee extension and flexion positions.

The most significant outcome of our research is the ability to describe the 3D anatomy of the ACL and its spatial position relative to the bone joint in a highly accurate manner that surpasses the methods currently used in orthopedic research. This capability opens up unprecedented opportunities to learn from nature at an unprecedented level of detail, leading to a deeper understanding of these structures and potential applications in solving engineering problems.

Future studies will focus on quantification analysis and correlation of these findings with the biomechanical properties of the ACL, exploring 3D microstructural properties even in pathological conditions. Such investigations have great potential to improve our understanding of ACL-related issues and could lead to advances in the diagnosis and treatment of knee joint pathologies.

### Supplementary Information

Below is the link to the electronic supplementary material.

Supplementary file1 (PDF 8 KB)

Supplementary file2 (MP4 29097 KB)

## Data Availability

The data that support the findings of this study are available from the corresponding author upon reasonable request.
