# Supplementary material for: 3D visualization of the human anterior cruciate ligament combining micro-CT and histological analysis
Source: Surg Radiol Anat. 2024 Jan 24;46(2):249–58. doi: 10.1007/s00276-023-03295-5 (PMC10861685; doi:10.1007/s00276-023-03295-5)
Supplement: Supplementary file 1 — Supplementary file1 (PDF 8 KB) [file 276_2023_3295_MOESM1_ESM.pdf]

Surgical and Radiologic Anatomy

3D visualization of the human Anterior Cruciate Ligament combining micro-CT and histological analysis

Annapaola Parrilli[1,*], Alberto Grassi[2,], Federica Orellana[1,3], Roberta Lolli[2], Gregorio Marchiori[2], Matteo Berni[2], Milena Fini[2], Nicola Francesco Lopomo[4], and Stefano Zaffagnini[2]

[1] Empa- Swiss Federal Laboratories for Materials Science and Technology, Dübendorf, Switzerland

[2] IRCCS - Istituto Ortopedico Rizzoli, Bologna, Italy

[3] University of Fribourg, Fribourg, Switzerland

[3] University of Brescia, Brescia, Italy

***Corresponding Author**

Dr. Annapaola Parrilli

Center for X-ray Analytics

Empa - Swiss Federal Laboratories for Materials Science and Technology

Überlandstrasse 129

8600 Dübendorf

Switzerland

email: annapaola.parrilli@empa.ch

Supplementary information

Caption

Video_1. 3D model of ACL anatomy. Some of the components of the knee joint have been gradually made transparent and the model has been virtually sectioned to describe the real development of the ACL fiber structure along its 3D path from the tibial to the femoral insertion. Bone is colored gray, cartilage is colored blue, ACL is colored orange, and PCL is colored yellow.
